# Albumin and hemoglobin adducts of estrogen quinone as biomarkers for early detection of breast cancer

**DOI:** 10.1371/journal.pone.0201241

**Published:** 2018-09-17

**Authors:** Po-Hsiung Lin, Hui-Ju Yang, Wei-Chung Hsieh, Che Lin, Ya-Chi Chan, Yu-Fen Wang, Yuan-Ting Yang, Kuo-Juei Lin, Li-Sheng Lin, Dar-Ren Chen

**Affiliations:** 1 Department of Environmental Engineering, National Chung Hsing University, South Dist., Taichung, Taiwan, R.O.C; 2 Department of Dermatology, Changhua Christian Hospital, Changhua, Taiwan, R.O.C; 3 Department of Internal Medicine, Da-Chien General Hospital, Miaoli, Taiwan, R.O.C; 4 Comprehensive Breast Cancer Center, Changhua Christian Hospital, Changhua, Taiwan, R.O.C; 5 Cancer Research Center, Department of Research, Changhua Christian Hospital, Changhua, Taiwan, R.O.C; 6 Department of Pharmacy, Changhua Christian Hospital, Changhua, Taiwan, R.O.C; 7 Department of Surgery, E-Da Hospital, I-Shou University, Kaohsiung, Taiwan, R.O.C; 8 Department of Breast Surgery, the Affiliated Hospital (Group) of Putian University, Putian, Fujian, China; 9 School of Medicine, Chung Shan Medical University, South Dist., Taichung, Taiwan, R.O.C; Nottingham Trent University, UNITED KINGDOM

## Abstract

Cumulative estrogen concentration is an important determinant of the risk of developing breast cancer. Estrogen carcinogenesis is attributed to the combination of receptor-driven mitogenesis and DNA damage induced by quinonoid metabolites of estrogen. The present study was focused on developing an improved breast cancer prediction model using estrogen quinone-protein adduct concentrations. Blood samples from 152 breast cancer patients and 71 healthy women were collected, and albumin (Alb) and hemoglobin (Hb) adducts of estrogen-3,4-quinone and estrogen-2,3-quinone were extracted and evaluated as potential biomarkers of breast cancer. A multilayer perceptron (MLP) was used as the predictor model and the resultant prediction of breast cancer was more accurate than other existing detection methods. A MLP using the logarithm of the concentrations of the estrogen quinone-derived adducts (four input nodes, 10 hidden nodes, and one output node) was used to predict breast cancer risk with accuracy close to 100% and area under curve (AUC) close to one. The AUC value of one showed that both data sets were separable. We conclude that Alb and Hb adducts of estrogen quinones are promising biomarkers for the early detection of breast cancer.

## Introduction

For more than half a century, early detection of breast cancer has been an important issue in cancer research. Pioneered by Egan [1] and then advanced by Wolberg & Mangasarian [2, 3], mammography is now a *de facto* technique for diagnosing the development of breast cancer and identifying the locations where a biopsy should be conducted. However, this technique relies heavily on visual inspection or computer aided pattern recognition techniques in order to recognize cancer cells. If cancer cells have not yet developed, diagnosis relies on biomarkers such as single nucleotide polymorphisms (SNPs) [4, 5], gene expression profiles [6], estrogen/progesterone receptors [7], DNA adducts [8], serum proteins [9, 10], and albumin and hemoglobin adducts of estrogen quinone [11, 12].

Cumulative estrogen concentration is an important determinant of the risk of developing breast cancer [13, 14]. Estrogen carcinogenesis is attributed to a combination of receptor-driven mitogenesis [15] and DNA damage induced by quinonoid metabolites of estrogen [16–19]. Metabolic activation of estrogen forms quinonoid metabolites that lead to the generation of promutagenic DNA lesions and the subsequent formation of oncogenic mutations derived from depurinating DNA adducts [20–23]. Cytochrome P450 1A1 and cytochrome P450 1B1 catalyze the oxidation of 17-estradiol (E_2_) to estrogen catechols including 2-hydroxyestradiol (2-OH-E_2_) and 4-hydroxyestradiol (4-OH-E_2_) [24–26]. Further conversion of 2-OH-E_2_ and 4-OH-E_2_ generates the respective estrogen quinones including estrogen-2,3-quinone (E_2_-2,3-Q) and estrogen-3,4-quinone (E_2_-3,4-Q) [27, 28]. These estrogen quinones are believed to play critical roles in the initiation of estrogen carcinogenesis [14, 18, 29].

In Taiwan, the onset of breast cancer tends to occur at a younger age than in western countries [30]. More than 50% of women diagnosed with breast cancers in Taiwan were found to be premenopausal [31] in contrast to approximately 25% of those in western populations. The incidence rate of breast cancer in Taiwanese women born after the 1960s is shifting toward that in Caucasian Americans. Environmental and dietary risk factors have been implicated in contributing to the increase in breast cancer in Taiwanese women. Development of biomarkers to identify individuals at high risk of developing breast cancer is a necessity. In our previous investigation [11, 12], we demonstrated that there are elevated levels of both the Alb and Hb adducts of E_2_-2,3-Q and E_2_-3,4-Q in breast cancer patients compared with those in healthy women. One of the unique features of this finding is that the ratio of Alb-E_2_-3,4-Q adducts to Alb-E_2_-2,3-Q adducts was 2:1 in breast cancer patients but 1:2 in healthy women, whereas the ratio of Hb-E_2_-3,4-Q adducts to Hb-E_2_-2,3-Q adducts in both breast cancer patients and healthy women was 2:1.

These recent findings suggest that Alb and Hb adducts of estrogen quinone could be used for early detection of breast cancer. A decision model could be developed based upon the concentration of the estrogen-quinone adducts. Visual inspection of scatter plots of the log-concentration values of E_2_-3,4-Q adducts versus E_2_-2,3-Q adducts of both Alb and Hb showed that the decision boundary of the model is unlikely to be a hyperplane. This suggested that a linear decision model such as logistic regression is not suitable for the decision model; instead, a non-linear decision model such as a multilayer perceptron would be more appropriate. In this report, we describe how a multilayer perceptron (MLP) with four input nodes, one hidden layer, and one output node could be applied to develop a decision model for breast cancer detection.

## Methods

### Data

The study population was recruited in a suburban medical center in central Taiwan. Women with breast cancer and healthy female subjects were recruited between May 2009 and May 2012. All the subjects provided sufficient venous blood for protein adduct analyses and completed questionnaires regarding age, body mass index, occupation, disease history, cigarette smoking, alcohol consumption, and dietary habits. Of those recruited, 152 breast cancer patients (BCP) and 71 healthy controls (HC) without any history of cancer were enrolled in the study. None of the enrolled individuals had a history of cancer, alcohol use, smoking, or chemotherapy. The age range of the BCP group was from 16 to 79 and the age range of the HC group was from 23 to 69. Mean age was 39.3 for HC and 50.8 for BCP. Of those recruited, 84 BCP and 58 HC were premenopausal. The Institutional Review Board of the Changhua Christian Hospital reviewed and approved this study (CCH IRB No. 081219). Each subject provided her written informed consent before participating in the study.

For each subject, the Alb adducts of E_2_-2,3-Q and E_2_-3,4-Q were analyzed from the serum following the procedure outlined in [11] and the Hb adducts of E_2_-2,3-Q and E_2_-3,4-Q were extracted from the red blood cells following the procedure outlined in [12]. All cysteinyl adducts arising from estrogen quinones were assayed using the procedure described previously [11]. Briefly, after bringing protein samples to complete dryness, estrogen quinone-derived adducts were cleaved after reaction with trifluoroacetic acid and methane sulfonic acid and analyzed via gas chromatography and mass spectrometry (S1 Data).

**Fig 1:**
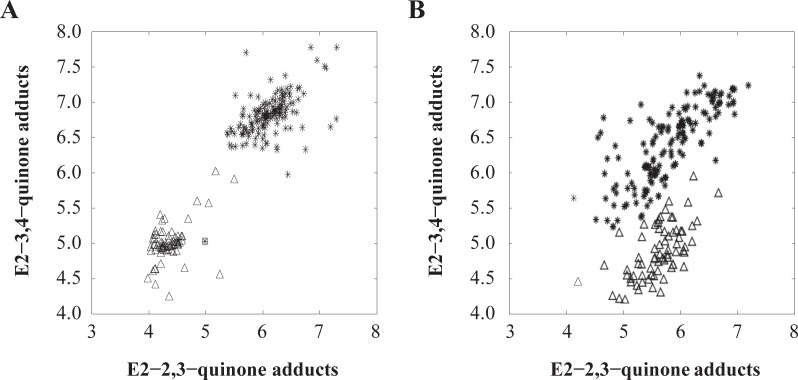
Scatter plots of the natural logarithm values of E_2_-3,4-Q and E_2_-2,3-Q adduct concentrations. (**A**) Hemoglobin adducts. (**B**) Albumin adducts. The asterisks (*) represent the cancer patients while the triangles (Δ) represent the healthy controls.

Fig 1 shows the scatter plots of the logarithm values of the E_2_-3,4-Q adduct concentrations plotted against those of the E_2_-2,3-Q adduct concentrations. Two observations are notable. First, models using Alb adducts or Hb adducts alone were unable to achieve 100% accuracy because both groups of data overlapped. Second, the concentration of Alb adducts of E_2_-3,4-Q was higher than that of E_2_-2,3-Q adducts in cancer patients with a ratio of approximately 2:1, whereas a ratio of 0.5 was observed in healthy controls, consistent with the finding in [31]. On the other hand, the levels of Hb adducts of E_2_-3,4-Q were higher than those of E_2_-2,3-Q adducts in both cancer patients and controls, with a ratio of approximately 2:1.

### Model

To construct the MLP model, we assumed that the training set $D=\{\left(x_{k},y_{k}\right){\}}_{k=1}^{223}$ was the set of blood samples obtained from the 223 subjects. Here, $x_{k}={\left(x_{k1},\cdots ,x_{k4}\right)}^{T}\in R^{4}$ was the *k*^*th*^ sample input and $Y_{k}\in \{0,1\}$ was the diagnostic result. If the *k*^*th*^ subject had cancer, $Y_{k}=1$. Else, $Y_{k}=0$. To model the risk function, we applied a MLP with four input nodes, 10 hidden nodes, and one output node. The inputs to the MLP were the logarithm values of the following adduct concentrations.

$$x_{k1}=\log(Conc.of\;Hb\;adducts\;of\;E_{2}‑3,4‑Q).$$

$$x_{k2}=\log(Conc.of\;Hb\;adducts\;of\;E_{2}‑2,3‑Q).$$

$$x_{k3}=\log(Conc.of\;Alb\;adducts\;of\;E_{2}‑3,4‑Q).$$

$$x_{k4}=\log\left(Conc.of\;Alb\;adducts\;of\;E_{2}‑2,3‑Q\right).$$

Let $w={\left(d^{T},c_{0},a_{1}^{T},\cdots ,a_{10}^{T},c_{1},\cdots ,c_{10}\right)}^{T}\in R^{61}$ be the parametric vector, where **d** ∈ *R*^10^ and *c*_0_ ∈ *R* are the weight vector and bias associated with the output node, respectively, and **a**_*j*_ ∈ *R*^4^ and *c*_*j*_ are the weight vector and bias associated with the *j*^*th*^ hidden node, respectively. The output of the MLP, *f* (**x**_k_,**w**), with input **x**_k_ is thus given by

$$f(x_{k},w)=\frac{1}{1+exp(-(\sum_{j=1}^{10}d_{j}h(x_{k},a_{j},c_{j})+c_{0}))}$$ (1)

where $h(x_{k},a_{j},c_{j})=1/\left(1+exp(-(\sum_{i=1}^{4}a_{ji}x_{ki}+c_{j}))\right)$ for *j =* 1,…, *m*.

To obtain the parametric vector **w**, we applied the gradient descent, minimizing the objective function given by

$$V(w)=\frac{1}{223}\sum_{k=1}^{223}{\left(y_{k}-f(x_{k},w)\right)}^{2}+\frac{\alpha }{2}{\left\|w\right\|}_{2,}^{2}$$ (2)

where the summation term is the mean squared error and the last term is the weight decay. The weight vector **w** is thus updated recursively by the following equation.

$$w\leftarrow w-\mu \nabla_{w}V(w),$$ (3)

where *μ* is the step size and ∇**w**V (**w**) is the gradient vector. In our study, *α* = 0.0004, *μ* = 0.02, and the total number of updates in (3) was 50000.

After the training was completed, the MLP was used to classify the samples. Let $y_{k}^{predict}$ be the class label of the *k*^*th*^ sample.

$$y_{k}^{predict}=\left\{ \begin{array}{l} \;1\;iff(x_{k},w)\geq t,\\ \;0\;otherwise, \end{array}\right.$$ (4)

where *t* ∈ [0, 1] is called the decision threshold. The *k*^*th*^ subject was classified as a cancer patient if $y_{k}^{predict}$ = 1. Otherwise, the *k*^*th*^ subject was classified as healthy. The training error rate was defined as the total misclassification over the size of training samples. Clearly, with different values of t, the error rate will be different. Usually, researchers arbitrarily set this value to 0.5.

In our analysis, we set the value of *t* in a different way. To determine the value of *t*, we attempted all the possible values of *t* from 0, 0.01, and so on, to 1. For each value of *t*, the training error rate was recorded. The value of *t* was then set to the one with the minimum training error rate and denoted as *t*_*opt*_. As shown in the analysis, there could have been a range of values that gave the same minimum training error rate if the MLP gave the same minimum training error rate, *t*_*opt*_ = (*t*_*min*_ *+ t*_*max*_) /2 for all t ∈ [tmin, tmax].

## Results

**Fig 2:**
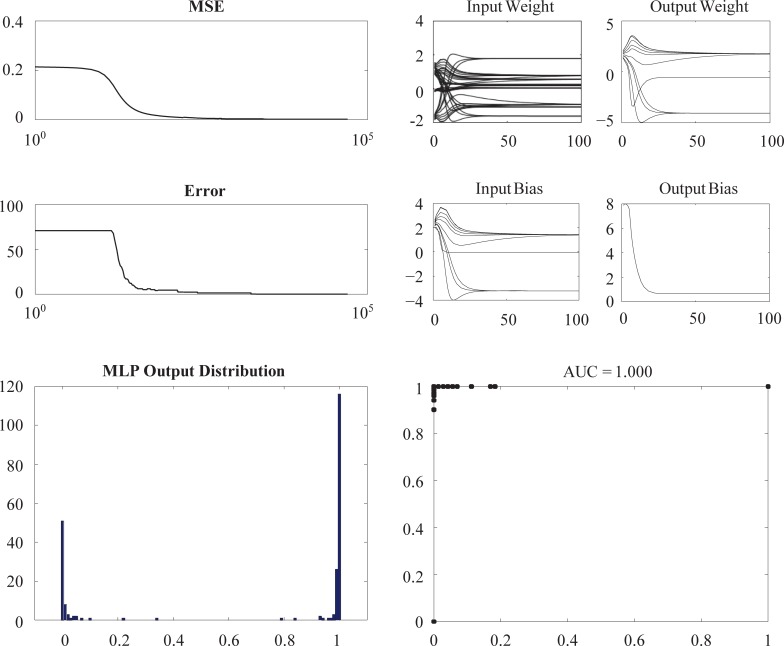
Typical training results.

Fig 2 shows the results of a typical training that used all four adducts of estrogen quinone. The results shown in the top panels indicate that the mean square error (MSE), the error rate (i.e. misclassification rate), and the parameters converged. Both the MSE and the error rate converged to zero. The right bottom panel shows that changing the threshold value *t* from 0 to 1 resulted in an area under curve (AUC) value of one. This indicated that the two sets of data were indeed separable.

Another four MLPs, each consisting of different combinations of three adducts, were generated based on the same procedure as for the MLP that used four adducts. The AUC analytical results are depicted in Table 1. For reference, the result of using all four adducts is included as Case 1. The results of Case 1, Case 2, and Case 5 revealed that HB adducts of E_2_-2,3-Q and Alb adducts of E_2_-3,4-Q are potential biomarkers for breast cancer detection. We also generated scatter plots of the logarithm values of the adduct concentrations; these demonstrated that while the two sets of data were separable, their set boundaries were very close to each other. Thus, MLPs generated using these two adducts may be sensitive to data error.

**Table 1:** AUC analysis for five combinations of adducts. All 223 samples (152 BCP samples and 71 HC samples) were used to obtain the MLP for prediction.

Case	Hemoglobin Adducts		Albumin Adducts		AUC
1	E_2_-3,4-Q	E_2_-2,3-Q	E_2_-3,4-Q	E_2_-2,3-Q	1.000
2	–	E_2_-2,3-Q	E_2_-3,4-Q	E_2_-2,3-Q	1.000
3	E_2_-3,4-Q	–	E_2_-3,4-Q	E_2_-2,3-Q	0.999
4	E_2_-3,4-Q	E_2_-2,3-Q	–	E_2_-2,3-Q	0.999
5	E_2_-3,4-Q	E_2_-2,3-Q	E_2_-3,4-Q	–	1.000

To validate the models, cross-validation was applied. Samples of BCP and HC were both randomly partitioned into two sets. The training set consisted of 80 percent of the samples (121 BCP samples and 56 HC samples) and the testing set consisted of the remaining samples. The MLP was trained using only the training set.

The AUC was obtained by changing the value of *t* from 0 to 1. The error rate and AUC were analyzed for both the training and testing sets. The process was repeated 20 times in all five cases as depicted in Table 1. Each time, a new training set was randomly generated. Five MLPs were obtained for the corresponding cases. For each MLP, the threshold value *t*_*opt*_ was obtained via the method described earlier. The average MSE, the average AUC, and their standard deviation values (shown inside parentheses) are shown in Table 2. These results showed that using the four adducts as biomarkers yielded superior accuracy in breast cancer detection compared with the results obtained using other biomarkers (Table 3).

**Table 2:** Validation results.

Case	Train Error (SD)	Test Error (SD)	Train AUC (SD)	Test AUC (SD)
1	0 (0)	0.0011 (0.0049)	1 (0)	1 (0)
2	0 (0)	0.0011 (0.0049)	1 (0)	1 (0)
3	0 (0)	0.0033 (0.0146)	1 (0)	0.9998 (0.0010)
4	0.0059 (0.0130)	0.0120 (0.0131)	0.9992 (0.0010)	0.9995 (0.0011)
5	0.0028 (0.0045)	0.0120 (0.0131)	0.9999 (0.0001)	0.9997 (0.0011)

SD, Standard error.

**Table 3:** Comparisons with previously reported results.

References	Biomarkers (No.)	Model	Test Error	AUC
This study	Adducts of estrogen quinone (4)	MLP	0.0011	1
[4]	SNP (1)	NB	0.33	–
[4]	SNP (2)	DT	0.32	–
[4]	SNP (3)	SVM	0.31	–
[9]	Serum proteins, Age, Race (100)	DF	–	0.84
[10]	Serum proteins (3)	BMA	0.15	0.82
[6]	Gene expression	PLSR	0.205	0.88
[32]	SNP from GWAS	KNN	0.3975	–
[33]	Gene expression (42)	SVM	–	0.7879
[5]	SNP (200)	SVM	0.0395	0.94
[34]	Mammogram	RF	0.0838	0.938
		NC	0.0859	0.962
		KNN	0.0644	0.967

BMA, Bayesian modeling averaging; DF, Data fusion; DT, Decision tress

GWAS, Genome-wide association study; KNN, K-nearest neighbors

MLP, Multilayer perceptron; NB, Niäve Bayes; NC, Nearest centroid

PLSR, Partial least square regression; RF, Random forest; SNP, Single nucleotide polymorphism

SVM, Support vector machine.

## Discussion

The high incidence rate of breast cancer in Taiwanese women emphasizes the need for better and more suitable screening and diagnostic technologies. In addition to the utility of mammography screening for early detection of breast cancer [1–3], recent studies have revealed the potential application of serum and plasma protein-based screening assays for diseases including prostate cancer, ovarian cancer, and breast cancer [10, 35, 36].

In this study, we aimed to develop a screening method with high sensitivity, specificity, and positive-predictive value for detecting breast cancer using blood protein adducts of estrogen quinones. Using MLPs, we were able to predict breast cancer risk based on the natural logarithm values of the estrogen quinone-protein adduct concentrations with an accuracy close to 100% and an AUC value close to one. The prediction results obtained using MLP with estrogen quinone-protein adducts were more accurate than those of other models [5, 6, 9, 10, 33]. In addition to the superior accuracy of our model compared with previously reported results of breast cancer prediction, the AUC value of one we obtained revealed that both data sets (cancer patients and healthy controls) were separable. Our findings strongly support the use of Alb and Hb adducts of estrogen quinone as biomarkers for early detection of breast cancer. These biomarkers can supplement the mammographic method in cases where cancer cells cannot yet be observed. However, the method presented in this investigation was developed using a retrospective study design. A prospective study using the above estrogen quinone-derived protein adducts would help validate these as biomarkers for early detection of breast cancer.

Taken together, this evidence lends further support to the idea that the cumulative concentration of estrogen quinone-protein adducts is a significant predictor of the risk of developing breast cancer. Further, the methodology developed in this study may also be applicable to other epidemiological studies and clinical trials in the prevention and early detection of breast cancer.

## Supporting information

S1 Data Quantitative analysis results of estrogen quinone-derived protein adducts. (XLSX)

## Abbreviations

Alb: Albumin
AUC: Area under curve
BMA: Bayesian modeling averaging
BCP: Breast cancer patient
DF: Data fusion
DT: Decision tress
E_2_-2,3-Q: Estrogen-2,3-quinone
E_2_-3,4-Q: Estrogen-3,4-quinone
GWAS: Genome-wide association study
Hb: Hemoglobin
HC: Healthy control
KNN: K-nearest neighbors
MLP: Multilayer perceptron
MSE: Mean square error
NB: Niäve Bayes
PLSR: Partial least square regression
SNP: Single nucleotide polymorphism
SVM: Support vector machine
